# Qianzheng powder for the treatment of primary Hemifacial spasm: A protocol for systematic reviews and meta-analysis

**DOI:** 10.1097/MD.0000000000025436

**Published:** 2021-04-09

**Authors:** Baoshan Chen, Rigun A, Ruizhen Yue, Guangrong Zhang

**Affiliations:** a Jiangxi University of Traditional Chinese Medicine, Nanchang, China; b Qihuang Outpatient Department of Jiangxi University of Traditional Chinese Medicine, Nanchang, China.

**Keywords:** primary Hemifacial spasm, protocol, Qianzheng powder, systematic review

## Abstract

**Background:**

Hemifacial spasm (HFS) is a clinical common neurological disease, its main performance for 1 side or 2 sides muscles (the orbicularis oculi muscle, expression, orbicularisoris muscles) recurrent paroxysmal, involuntary twitching, aggravating when excited or nervous, more severe cases of the disease may include difficulty in opening the eyes, crooked corners of the mouth, and twitching noises in the ears, etc.^[1]^ Early manifestations of the disease are intermittent mild convulsions of the orbicularis oculi muscle, and then gradually spread to 1 side of the facial muscles, such as frowning muscles, nasal muscles, buccinalis muscles, etc, especially the most obvious spasms of the oral muscles, which can involve the ipsilateral platysma muscle in severe cases, with each twitch for a few seconds to a few minutes. The disease will affect the quality of life such as speaking, eating, seeing and so on, and even cause psychological effects such as inferiority, anxiety and depression. At present, the incidence of the disease in China is 11 per 1.1 million, females are more common than males. There are many ways to treat HFS, but the Qianzheng powder has a unique advantage in treating this disease. Therefore, our systematic review aims to evaluate the efficacy and safety of Qianzheng powder in the treatment of Primary Hemifacial spasm, and to provide a reliable basis for clinical decision makers.

**Methods:**

From its inception until April 2021, we will search electronic databases, including PubMed, Embase, Cochrane Library, China Biomedical Literature Database, China Knowledge Infrastructure, Wanfang Database, and China Scientific Journals Database. The authors will independently sift through studies, extract data information, and assess methodological quality using the Cochrane Risk of Bias tool. The RevManV. 5.3 software will be used for statistical analysis.

**Results:**

The results of this study, which will be published in a peerreviewed journal, will evaluate the efficacy and safety of Qianzheng powder in the treatment of primary Hemifacial spasm.

**Conclusion:**

This systematic review will provide reliable evidence-based basis for treating primary Hemifacial spasm with Qianzheng powder.

**INPLASY Registration number:**

INPLASY202130037.

## 1 Introduction

Primary Hemifacial spasm is a common benign functional disease in clinic. The main clinical manifestations are involuntary, paroxysmal, recurrent twitching of unilateral facial muscles. Mental tension, anxiety and too much psychological pressure can induce or aggravate the attack. Movements of facial muscles, such as forcibly closing the eyes or bulging the gills, can also trigger spasm, even during sleep and under anesthesia. Typical HFS refers to the initial onset of spasm of the orbicularis oculi muscle, and then the progression downward, gradually involving the cheek, the horns of the mouth, and the platysma muscle. Atypical HFS is characterized by the initial location of the perioral muscle, and then from the bottom up to involve half of the facial muscles.^[2]^ In clinical atypical HFS is less, the vast majority are typical HFS.^[3–5]^ HFS usually occurs in middle and old age, females are more common than males, however, the age of onset tends to be younger.^[6]^ Bilateral HFS are not uncommon, although they are mostly on 1 side.^[7,8]^ At present, the etiology and pathogenesis of primary HFS have not been fully understood in modern medicine. Vascular factor is considered to be the most common in clinical, it is facial nerve is oppressed by artery blood vessel for a long time in REZ then facial nerve myelin sheath is destroyed, and then it leads to a short circuit between the afferent and efferent nerve fibers, resulting in spasm. Treatment for primary HFS includes oral antiepileptic and sedative drugs, botulinum toxin injections, and surgery. Oral drugs are not good, side effects are large; Ineffectiveness, recurrence and a series of complications after the operation are still a major problem. Chinese medicine is an important part of TCM therapy and has been practiced in China for thousands of years. Traditional Chinese medicine believes that the etiology and mechanism of the disease is the external attack of wind pathogen and the obstruction of collaterals by phlegm and blood stasis. Wind pathogen is the external cause of HFS, and phlegm and blood stasis play a key role in the pathogenesis of HFS.

The giant typhonium rhizome in Qianzheng powder is pungent and warm-dryness drug. It enters Yangming meridian and goes to the head to dispel pathogenic wind and eliminate phlegm, especially good for the wind of the head. Scorpion and stiff silkworm can dispel pathogenic wind and stop spasms, among which the scorpion is good at removing obstruction in collaterals, and the stiff silkworm can eliminate phlegm. And mixing with hot wine can promote blood circulation and lead medicine into the collaterals and direct to the lesion site. The combination of all the drugs can not only dispel pathogenic wind and eliminate phlegm, but also remove obstruction in collaterals and stop spasms.^[9]^ Modern clinical studies have shown that the possible mechanism of Qianzheng powder's treatment of HFS is that the active ingredients of many drugs in it play a role through multi-target, multi-system and multi-aspect, reducing inflammation, accelerating cell repair, dilating blood vessels, improving microcirculation, accelerating nerve repair and alleviating smooth muscle spasm.^[10]^ Thus, it can effectively improve the spastic intensity of patients with HFS, improve the quality of life of patients and the effect is definite and lasting. However, there is still a lack of systematic review and meta-analysis on the efficacy and safety of Qianzheng powder in the treatment of HFS. Therefore, we will conduct a systematic review and meta-analysis of the efficacy and safety of clinical randomized controlled trials of Qianzheng powder in the treatment of primary HFS in order to provide reliable evidence for clinical decision makers.

## 2 Methods

### 2.1 Inclusion criteria

#### 2.1.1 Types of studies

Randomised controlled trials evaluating Qianzheng powder in the treatment of primary HFS will be eligible for inclusion and will be published in English or Chinese, with the full-text available.

#### 2.1.2 Types of participants

Patients with primary HFS with a clear diagnostic criteria will be included. The study was not limited to race, age, sex, nationality, and source of cases. Participants with secondary HFS will be excluded.

#### 2.1.3 Types of interventions

Qianzheng powder as a single intervention means, there is no limit to the dosage, addition or subtraction of the medication, or in combination with other intervention measures (such as conventional drugs, acupuncture, moxibustion, etc.).

#### 2.1.4 Types of control group

The control group had no limits on treatment, including conventional drugs, no treatment, or placebo.

#### 2.1.5 Types of outcome measures

##### 2.1.5.1 Primary outcome

Total effective rate (assessed based on Cohen criteria for efficacy evaluation of Hemifacial spasm, total effective rate = (cure + significant effect + effective)/total).

##### 2.1.5.2 Additional outcomes

Adverse events will be considered as additional consequences.

### 2.2 Search methods for the identification of studies

We will search electronic databases, including PubMed, Embase, Cochrane Library, China Biomedical Literature Database, China National Knowledge Infrastructure, Wanfang Database, and China Science Journals Database, to collect potential randomized controlled trials (RCTs) from the start through April 2021.

Search terms of disease: HFS, primary HFS, neuralgica chorea, facial muscles twitch; and intervention: Qianzheng powder, Chinese medicine; and type of research: randomized controlled trial, clinical controlled trial, randomized. The PubMed search strategy is shown in Table 1.

**Table 1:** Search strategy (PubMed database).

Number	Search items
#1	Mesh: “Hemifacial spasm”
#2	Ti/Ab: “Hemifacial spasm” OR “Primary Hemifacial spasm” OR “Spasm, Hemifacial” OR “Hemifacial Spasms” OR “Spasms, Hemifacial” OR “Facial Spasm, Unilateral” OR “Facial Spasms, Unilateral” OR “Spasm, Unilateral Facial” OR “Spasms, Unilateral Facial” OR “Unilateral Facial Spasm” OR “Unilateral Facial Spasms” OR “Hemifacial Myokymia” OR “Myokymia, Hemifacial”
#3	#1 OR #2
#4	Mesh: “Drugs, Chinese Herbal”
#5	Ti/Ab: “Drugs, Chinese Herbal” OR “Qianzheng powder” OR “Chinese Drugs, Plant” OR “Chinese Herbal Drugs” OR “Herbal Drugs, Chinese” OR “Plant Extracts, Chinese” OR “Chinese Plant Extracts” OR “Extracts, Chinese Plant”
#6	#4 OR #5
#7	Mesh: “randomized controlled trial [Publication Type]” OR “randomized controlled trials as topic” OR “controlled clinical trial [Publication Type]” OR “controlled clinical trials as topic”
#8	Ti/Ab: “randomized”
#9	#7 OR #8
#10	#3 AND #6 AND #9

### 2.3 Data collection and analysis

#### 2.3.1 Selection of studies

**Figure 1:**
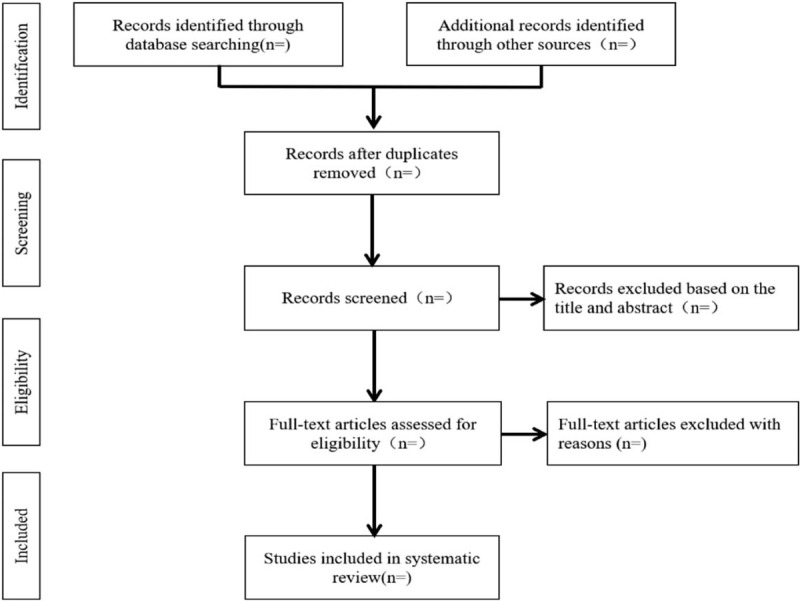
Flow diagram of study selection process.

We will import the retrieved results into the endnote X7 software and remove the duplicated data. After that, the authors will independently scan the titles, abstracts, and full texts in accordance with inclusion and exclusion criteria to assess the eligibility of the articles. Any disagreement will be resolved by the third author. The study selection procedure is summarized in Figure 1.

#### 2.3.2 Data extraction and management

Two reviewers will independently extract relevant data from eligible RCTs, including first author, baseline characteristics of participants, sample size, intervention, duration of intervention, follow-up, outcomes, and adverse events. Any discrepancies will be resolved by consulting a third-party reviewer. If necessary, we will also contact the original author for more information.

### 2.4 Risk of bias assessment

The bias risk assessment tool recommended by the Cochrane Collaboration Network was used to assess the quality of the included studies. The following 7 evaluation items are included:

1. random sequence generation;
2. allocation concealment;
3. blinding of participants and personnel;
4. blinding of outcome assessment;
5. incomplete outcome data;
6. selective reporting;
7. other sources of bias

For each study, the results of the 7 items were rated as “Yes” (low risk), “No” (high risk), and “unclear” (lack of information or uncertainty about bias). Two reviewers independently conduct quality assessments and any differences will be resolved through discussion.

### 2.5 Quantitative data synthesis and statistical methods

#### 2.5.1 Quantitative data synthesis

We will use RevMan V. 5.3 software for statistical analysis. For dichotomous variables, relative risk (RR) and 95% confidence intervals (CI) were used.

#### 2.5.2 Assessment of heterogeneity

We will use the *I*^2^ test and the Chi-Squared test to evaluate the heterogeneity of the results. When *I*^2^ < 50% and *P* > .10, the results will be considered homogeneous and the fixed-effect model will be used. Otherwise, the random effects model is used.

#### 2.5.3 Subgroup analysis

If significant heterogeneity is detected in our meta-analysis, we will perform subgroup analysis according to different control groups.

#### 2.5.4 Sensitivity analysis

When sufficient RCTs are available, we will conduct sensitivity analysis based on method quality, sample size, and missing data to assess the robustness of the study results.

#### 2.5.5 Assessment of reporting biases

Publications bias will be analyzed through the funnel plots. If the funnel plot is asymmetric, there may be publication bias in the results.

## 3 Discussion

This study will systematically review and meta-analyze the efficacy and safety of Qianzheng powder in the treatment of primary HFS. As far as we know, this will be the first study in this area. The conclusions of this study can provide evidence-based medicine suggestions for treating primary HFS with Qianzheng powder, and provide more and better treatment options for patients with primary HFS. However, our conclusions may have some potential limitations. First, different drug doses, drug addition and subversion, severity of the disease, duration of treatment, and different control groups may lead to potential heterogeneity. Heterogeneity must be explained by subgroup analysis or sensitivity analysis. Second, the measurements and tools for the results of included RCTs may differ.

## Author contributions

**Data curation:** Baoshan Chen, Rigun A.

**Formal analysis:** Baoshan Chen, Rigun A.

**Investigation:** Baoshan Chen, Rigun A.

**Methodology:** Rigun A, Ruizhen Yue.

**Project administration:** Baoshan Chen, Guangrong Zhang.

**Software:** Rigun A, Ruizhen Yue.

**Supervision:** Guangrong Zhang.

**Validation:** Guangrong Zhang.

**Writing – original draft:** Baoshan Chen, Guangrong Zhang.

**Writing – review & editing:** Baoshan Chen, Guangrong Zhang.
